# Financial Conflicts of Interest in Propensity Score-Matched Studies Evaluating Biologics and Biosimilars for Inflammatory Bowel Disease

**DOI:** 10.1093/jcag/gwac018

**Published:** 2022-06-01

**Authors:** Karam Elsolh, Daniel Tham, Michael A Scaffidi, Nikko Gimpaya, Rishi Bansal, Nazi Torabi, Juana Li, Yash Verma, Rishad Khan, Samir C Grover

**Affiliations:** Michael G. DeGroote School of Medicine, McMaster University, Hamilton, Ontario, Canada; Division of Gastroenterology, St. Michael’s Hospital, University of Toronto, Toronto, Ontario, Canada; Division of Gastroenterology, St. Michael’s Hospital, University of Toronto, Toronto, Ontario, Canada; Division of Gastroenterology, St. Michael’s Hospital, University of Toronto, Toronto, Ontario, Canada; Faculty of Health Sciences, School of Medicine, Queen’s University, Kingston, Ontario, Canada; Division of Gastroenterology, St. Michael’s Hospital, University of Toronto, Toronto, Ontario, Canada; Michael G. DeGroote School of Medicine, McMaster University, Hamilton, Ontario, Canada; Division of Gastroenterology, St. Michael’s Hospital, University of Toronto, Toronto, Ontario, Canada; Li Ka Shing Knowledge Institute, Toronto, Ontario, Canada; Division of Gastroenterology, St. Michael’s Hospital, University of Toronto, Toronto, Ontario, Canada; Division of Gastroenterology, St. Michael’s Hospital, University of Toronto, Toronto, Ontario, Canada; Division of Gastroenterology, St. Michael’s Hospital, University of Toronto, Toronto, Ontario, Canada; Department of Medicine, University of Toronto, Toronto, Ontario, Canada; Division of Gastroenterology, St. Michael’s Hospital, University of Toronto, Toronto, Ontario, Canada; Li Ka Shing Knowledge Institute, Toronto, Ontario, Canada; Department of Medicine, University of Toronto, Toronto, Ontario, Canada

**Keywords:** Biologic drugs, Biosimilar pharmaceuticals, Conflict of Interest, Inflammatory bowel disease, Propensity score

## Abstract

**Background:**

Propensity score matching (PSM), a statistical technique that estimates a treatment effect by accounting for predictor covariates, has been used to evaluate biologics for inflammatory bowel disease (IBD). Financial conflicts of interest are prevalent in the marketing of biologic medications. It is unclear whether this burden of conflicts is present among authors of PSM studies comparing IBD biologics and biosimilars.

**Objective:**

This study was aimed to determine the prevalence of financial conflicts of interest among authors of PSM studies evaluating IBD biologics and biosimilars.

**Methods:**

We conducted a systematic search for PSM studies comparing biologics and biosimilars in IBD treatment. We identified 21 eligible studies. Two independent authors extracted self-declared conflicts from the disclosures section. Each participating author was searched on the Centers for Medicare & Medicaid Services Open Payments to identify payment amounts and undisclosed conflicts. Primary outcome was the prevalence of author conflicts. Secondary analyses assessed for an association between conflict prevalence and reporting of positive outcomes.

**Results:**

Among 283 authors, conflicts were present among 41.0% (116 of 283). Twenty-three per cent (27 of 116) of author conflicts involved undisclosed payments. Studies with positive outcomes were significantly more likely to include conflicted authors than neutral studies (relative risk = 2.34, 95% confidence interval: 1.71 to 3.21, *P* < 0.001).

**Conclusions:**

Overall, we found a high burden of undisclosed conflicts among authors of PSM studies comparing IBD biologics and biosimilars. Given the importance of PSM studies as a means for biologic comparison and the potential for undue industry influence from these payments, authors should ensure greater transparency with reporting of industry relationships.

## INTRODUCTION

The emergence of multiple biologics and biosimilars to manage inflammatory bowel disease (IBD) has necessitated a comprehensive approach when deciding on a therapeutic regimen (1–3). One approach to this issue is to examine real-world data of competing biologics using propensity score matching (PSM). PSM is a statistical technique that estimates a treatment effect by accounting for baseline patient characteristics (4). The results of PSM studies are becoming influential in evidence-based planning of IBD treatment, wherein several of these studies have been cited by clinical practice guidelines (CPGs) (5, 6). Of concern, however, is the potential for undue influence from financial conflicts of interest (FCOI) among research of biologics in IBD (7). The role of FCOI among these studies has not been investigated.

Financial conflicts are prevalent in medical literature and can influence clinical decision making (8–10). The extent of this influence has been elucidated by a number of studies. For example, a 1998 study by Stelfox et al. demonstrated a strong association between authors’ published positions on the safety of calcium channel blockers and their financial relationships with pharmaceutical manufacturers (11). Additionally, industry-sponsored studies have been shown to yield favourable results for the sponsors (12).

Specific to IBD, industry payments are prevalent in the marketing of biologic medications and in point-of-care resources for IBD management (7, 13, 14). A substantial portion of guideline panels of IBD CPGs, for instance, have been found to have FCOI (15, 16). Furthermore, these FCOI may, in fact, significantly influence physicians’ treatment recommendations, as suggested by a study linking industry payments from biologic manufacturers and physicians’ prescribing of these drugs (17).

To date, no study has investigated the prevalence and impact of FCOI among PSM studies evaluating IBD biologics. PSM studies may be particularly susceptible to such FCOI because they compare multiple medications head-to-head, a phenomenon that has been shown to raise the incidence of FCOI in randomized controlled trials (RCTs) (18). Unlike RCTs which are expensive (and consequently essentially require payments from sponsors to execute), PSM cohort studies can be performed without industry payments to physicians, resulting in the need to further characterize potential conflicts arising from such payments. This is of particular relevance as PSM studies are increasingly being used to guide clinical recommendations and have been cited by CPGs, yet conflicts in these studies to date have not been scrutinized to the same degree as those in RCTs (5, 6). In this study, we determined the prevalence of FCOI among propensity score-matched studies evaluating IBD biologics and biosimilars, and association of FCOI with positive study outcomes.

## METHODS

We systematically evaluated PSM studies that compared real-world data of biologic/biosimilar effectiveness in the management of Crohn’s disease and ulcerative colitis for FCOI. Reporting of our findings followed guidelines for the reporting of meta-epidemiological methodology research (adapted PRISMA statement) (19).

### Definitions

We defined ‘relevant FCOI’ as payments made to authors from pharmaceutical manufacturers of the biologic and/or biosimilar investigated in the PSM study (14). We used the term ‘author’ to describe each instance of a contributor appearing in a PSM study. We categorized FCOI as either disclosed or undisclosed. Disclosed FCOI were defined as relevant payments listed by authors in the disclosure section of the publication. Undisclosed FCOI included any additional, relevant payments we identified through the Centers for Medicare & Medicaid Services Open Payments (CMS-OP) Database that were not included in the article’s disclosure section. We considered a study to use PSM if study participants in one group were matched to participants in a comparison group on the basis of propensity score, which is a summary score of patients’ baseline characteristics (20). We defined study methodologists as authors responsible for study design and methodology, as identified in the author contributions declaration of the study. Outcomes were considered ‘positive’ if a study had at least one statistically significant primary outcome (evaluated at the level defined by the study authors) in which a biologic/biosimilar was found to be more effective than the comparator (21). Non-inferiority studies were excluded from this definition. Concordance was defined as a correspondence, assessed statistically, between conflict payments and the favoured drug companies.

### Study Selection

We conducted a systematic search on October 2020 on EMBASE (Ovid), Medline (Ovid) and Cochrane Library (see Supplementary Tables 1–3, which outline the full search strategy). The search was designed by a librarian. We only included records published in English. Any duplicated records were manually removed during screening. Two authors (K.E. and D.T.) independently reviewed the abstracts and full texts for eligible studies, wherein any discrepancies were resolved by consensus. We included studies that met the following criteria: use of PSM to compare either biologics-to-biologics or biologics-to-biosimilars; enrolment of patients with IBD (Crohn’s disease, ulcerative colitis or indeterminate colitis); and evaluation of therapeutic effectiveness, efficacy or safety as primary endpoints. We excluded research letters, conference presentations, editorials, commentaries and abstract-only submissions.

### Databases

To determine industry payments made to authors of included studies, we used data from the CMS-OP database. CMS-OP is a publicly accessible database that catalogues financial payments from industry manufacturers/group purchasing organizations to US physicians (22).

### Data Extraction

From each full text, two authors (K.E. and D.T.) independently obtained the following data in duplicate: full list of authors, with associated position in authorship; author study contributions (e.g., study design, data acquisition); names of included biologics and/or biosimilars; industry manufacturers of the biologics and/or biosimilar agents; and declared FCOI for each participating author from the study disclosures section.

These same two authors also extracted the following data, when available, from CMS-OP for any authors included in the database: presence of any undisclosed FCOI, and payment amounts for both disclosed and undisclosed FCOI. In particular, we obtained payment data from CMS-OP for 3 years up to and including the year of study publication in accordance with the standardized disclosure recommendations of the *International Committee of Medical Journal* Editors (ICMJE) (22). Both disclosed and undisclosed FCOI were classified by payment type into one of three categories: general, research or ownership/investment, in accordance with the classification system from CMS-OP (23). Payment amounts are reported in USD.

### Outcome Measures

The primary outcome was the overall prevalence of author FCOI among propensity score-matched studies evaluating biologics and/or biosimilars for IBD. We stratified the prevalence of FCOI by disclosure status and payment type. Secondary analyses were exploratory and investigated the following: the association of FCOI prevalence with positive outcomes; concordance of FCOI received with the pharmaceutical producer favoured in the study; whether FCOI prevalence differed by authorship position (first author versus middle author versus last author); and whether FCOI prevalence differed by type of comparison conducted (biologic/biologic versus biologic/biosimilar). We conducted an subgroup analysis to investigate the association of FCOI prevalence with author methodologist status.

### Statistical Analysis

All statistical analyses were conducted using both R (v. 1.2.5019) and SPSS (v. 27, SPSS Inc., Armonk, NY). Quantitative and categorical variables were presented as means with standard deviation (SD) (or median with interquartile range [IQR]) and as count with percentages, respectively. We calculated FCOI prevalence as the proportion authors with at least one instance of relevant FCOI among all authors involved in the included studies. We used Fisher’s exact test and chi-squared tests to determine whether there was an association between FCOI prevalence with positive study outcomes, an association between FCOI prevalence and author methodologist status, between FCOI prevalence and authorship position, between FCOI prevalence and the type of comparison conducted, and whether there was a concordance between the presence of at least one FCOI and the favoured drug companies. We used two-sample unpaired *t*-tests to evaluate for concordance between dollar amount of FCOI and the favoured drug companies, including a sensitivity analysis of a subgroup of 20 authors for whom payment data were available on CMS-OP. All statistical tests were two-tailed and considered significant at *P* <0.05 using exact *P*-values. RR represents relative risk, 95% CI represents 95% confidence interval and $\bar{x}$ represents mean value.

## RESULTS

**Figure 1.**
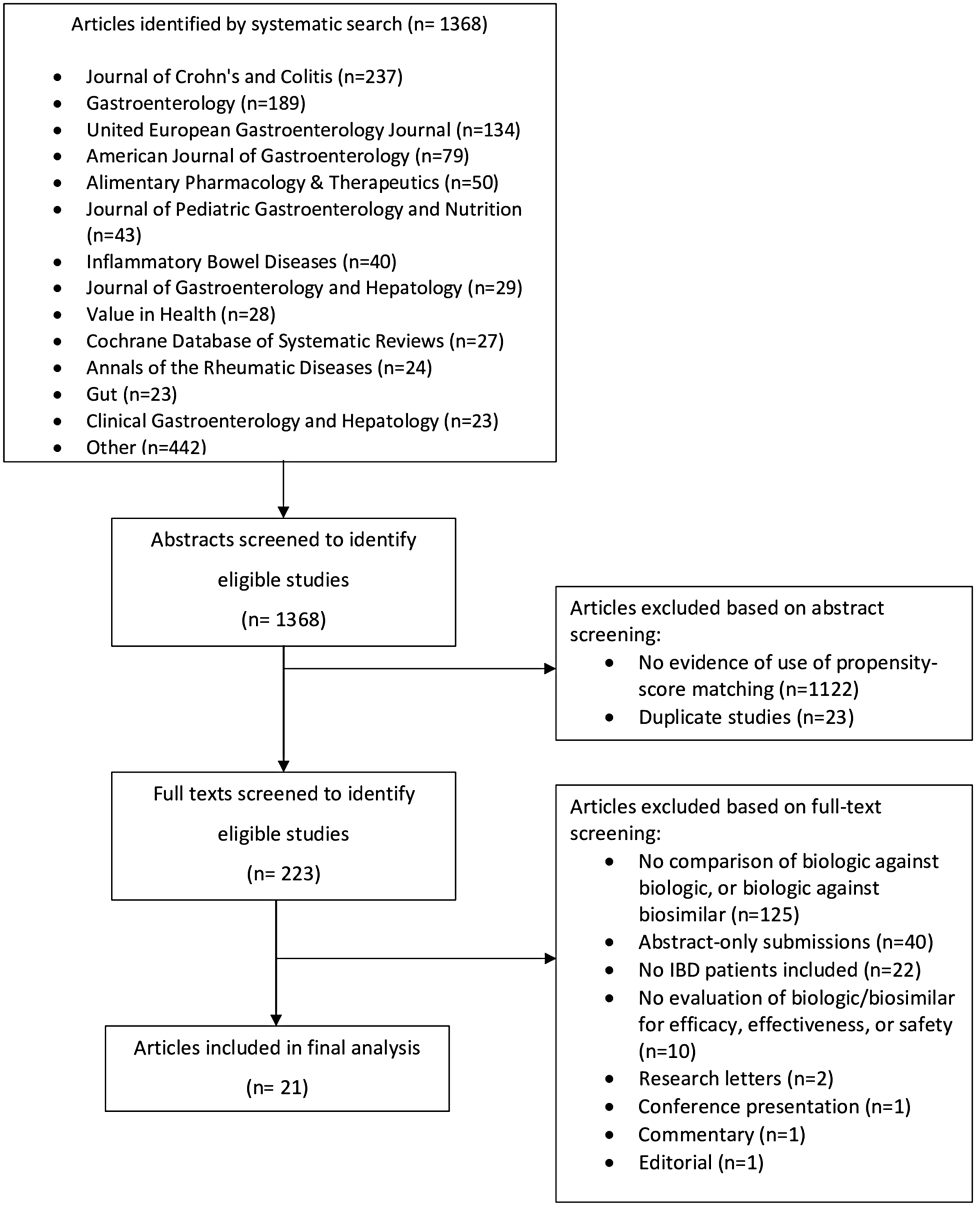
Study flow chart.

Our search strategy identified an initial 1,368 articles (Figure 1). Following both screening and full-text review, we included a total of 21 articles for analysis with 283 authors. Most authors (247, 87.3%) were affiliated with an academic institution, 83.4% (236) were physicians, and 40.3% (114) were based in U.S. institutions. A summary of author characteristics can be found in Table 1.

**Table 1.** Author characteristics (*n* = 283)

Characteristic	*n* (%)
Physician	
Yes	236 (83.4)
No	47 (16.6)
Author institution (type)	
Academic institution	247 (87.3)
Governmental organization	10 (3.5)
Community health centre	23 (8.1)
Private practice	3 (1.1)
Author institution (location)	
United States	114 (40.3)
Italy	79 (27.9)
France	33 (11.7)
Spain	25 (8.8)
The Netherlands	16 (5.6)
Denmark	15 (5.3)
Canada	1 (0.4)

### Prevalence of FCOI

**Figure 2.**
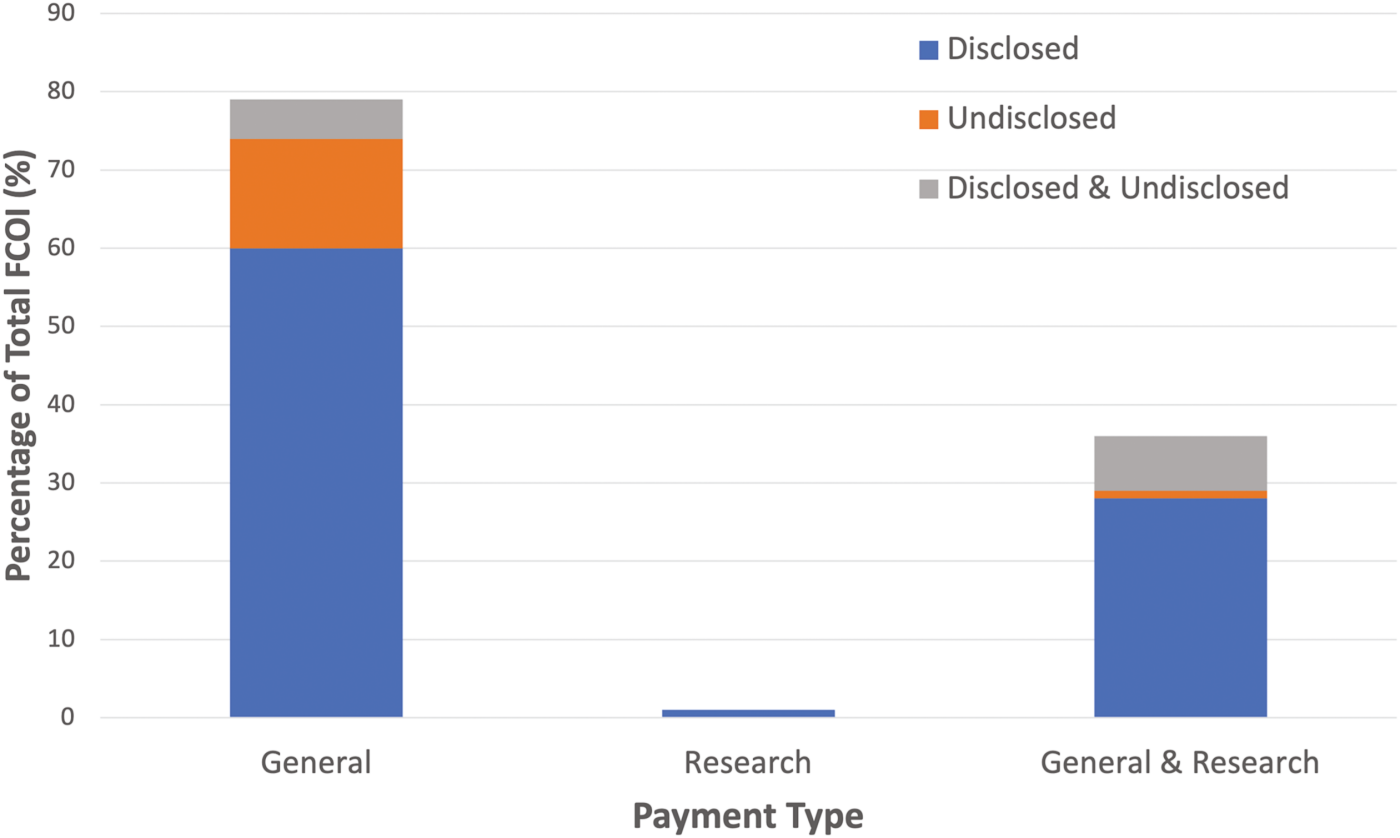
Proportion of author FCOI by payment type and disclosure status. FCOI, Financial conflict of interest.

A summary of FCOI prevalence by payment type and disclosure status is provided in Figure 2. We found that prevalence of authors with at least one relevant FCOI was 41.0% (*n =* 116). Among the 116 authors with relevant FCOI, 76.7% (*n =* 89) fully disclosed their relevant payments, 12.9% (*n =* 15) did not disclose any of their payments and the remaining 10.4% (*n =* 12) had both disclosed and undisclosed FCOI. Among these authors with relevant FCOI, 68.1% (*n =* 79) received general payments only, 0.8% (*n =* 1) received research payments only, 31.0% (*n =* 36) received both general and research payments and none received payment in the form of equity.

Of 283 authors, 48 had CMS-OP accounts with payment data publicly available. We found a total dollar value of FCOI of $1,612,139.0 ($1,604,367.10 as general payments, $7,771.90 as research payments), with a median FCOI value of $12,124.70 (IQR: $690.50 to $62,558.60). The median dollar value was $8,917.70 (IQR: $690.50 to $61,135.50) for general payments and $1,883.90 (IQR: $303.70 to $4,817.50) for research payments. Overall, the median dollar value for undisclosed FCOI was $697.10 (IQR: $170.40 to $1,352.50) among 48 authors with CMS-OP accounts.

When considered at a study level, 90.5% (*n =* 19) of studies had at least one instance of an author with relevant FCOI. Furthermore, 85.7% (*n* = 18) of studies had at least one author with a disclosed FCOI and 42.9% (*n* = 9) of studies had at least one author with an undisclosed FCOI. Among studies with disclosed FCOI, a mean of 39.9% (SD = 25.0%) of authors per study reported relevant FCOI. Among studies with undisclosed FCOI, a mean of 21.8% (SD = 13.0%) of authors per study reported relevant FCOI.

### Secondary Outcomes

A summary of the secondary analyses is provided in Table 2. We found that studies with positive outcomes were significantly more likely to include conflicted authors than neutral studies (RR = 2.34, 95% CI: 1.71 to 3.21, *P* < 0.001). Among nine studies that favoured a particular biologic/biosimilar, authors were no more likely to receive at least one relevant FCOI from the favoured company than from the comparator ($\bar{x}$ = 0.88 versus $\bar{x}$ = 0.92, *t* = 0.81, *P* = 0.42). We did not find a significant concordance between the dollar amount of FCOI and the favoured company relative to the comparator ($\bar{x}$ = $9,185.5 versus $\bar{x}$ = $5,350.8, *t* = 0.97, *P* = 0.33). A sensitivity analysis of 20 authors for whom payment data were available did not demonstrate a significant concordance between FCOI in dollar amount and the favoured company relative to the comparator ($\bar{x}$ = $34,787.8 versus $\bar{x}$ = $20,808.6, *t* = 1.07, *P* = 0.29). There was no significant difference in prevalence of relevant FCOI between studies comparing biologics to biologics (*n* = 20) and a study that compared switching from a biologic to a biosimilar (*n* = 1) (RR = 0.70, 95% CI: 0.48 to 1.02, *P* = 0.22). In addition, there was no significant association between authorship position and presence of relevant FCOI (*P* = 0.45), or between methodologist status and presence of FCOI (RR = 0.99, 95% CI: 0.75 to 1.30, *P* = 0.93) among authors with published study contributions.

**Table 2.** Association between author FCOI and primary outcomes, type of comparison conducted

	Primary outcome		Type of comparison	
	Statistically significant	Neutral	Biologic vs. biologic	Biologic vs. biosimilar
Total # of authors	143	140	277	6
FCOI present	84 (49.0%)	32 (22.9%)	115 (41.5%)	1 (16.7%)
FCOI absent	59 (51.0%)	108 (77.1%)	162 (58.5%)	5 (83.3%)
RR (95% CI)	2.34 (1.71, 3.21)		0.70 (0.48, 1.02)	
*P*-value	<0.001		0.22	

FCOI, Financial conflict of interest; RR, relative risk; 95% CI, 95% confidence interval. *P*-value <0.05 considered significant

## DISCUSSION

In our analysis of 21 studies using PSM that compared biologics and/or biosimilars for the management of IBD, we found that approximately 41% of all authors had relevant FCOI with a median payment value of $12,124.70 among 48 authors with CMS-OP accounts. Twenty-three per cent of authors had undisclosed payments. Studies with positive outcomes were more likely to include conflict payments than neutral studies.

Our findings, which are consistent with prior literature that also found a high prevalence of FCOI in IBD CPGs, may be explained by several factors (4). In general, financial relationships between gastroenterologists and biologic manufacturers are highly prevalent, with a previous study finding that 99% of U.S. physicians with CMS-OP accounts had a financial relationship with a biologic manufacturer (17). There may be more payments related to biologics as they are relatively new and high-grossing medications and have been associated with informational meetings, meals and physician talks on behalf of pharmaceutical companies (24). Accordingly, these manufacturers consult on extensive physician marketing campaigns (24). A 2010 report summarizing spending by top pharmaceutical companies cited that the companies producing adalimumab and infliximab spent 42.2 million USD and 6.3 million USD, respectively, on direct-to-physician marketing (24).

In addition to the high prevalence of FCOI, our findings also highlight a link between industry relationships and the results of PSM studies, as studies with positive outcomes had a higher likelihood of relevant author FCOI than neutral studies. This finding is consistent with work done in rheumatology, which has demonstrated an independent association of author consulting fees and positive RCT outcomes (25). Our results may raise concern for the possible impact of FCOI on the reporting of study results. However, this association did not appear to have a directionality, as we did not find a concordance between FCOI and the drug company that was favoured either in number or in dollar amount. Furthermore, several authors received FCOI from both the favoured drug company and the comparator. FCOI were not significantly in concordance with study outcomes as authors may have FCOI with both arms of the study. We do not know at this time the effects of FCOI on study results but a regulatory standardized process to deal with FCOI is hoped for. FCOI may introduce bias to a study but are limited as a quantitative metric in capturing the role of bias in the design, conception and execution of a study and the reporting of its results (26). Because of this, along with the potential for conflict omission, the impact of FCOI is difficult to tease out from other sources of bias. Nevertheless, PSM studies are an important form of comparative evidence for clinicians and several in our cohort have been cited by CPGs in IBD management (5, 6). Ensuring objectivity and minimizing bias in these studies remains an important goal.

We note several study limitations. First, our use of the CMS-OP database restricted categorization of undisclosed payments to U.S.-based physicians only, which likely underestimated our reported prevalence of undisclosed FCOI and payment amounts. In addition, one of the 21 included studies was published in 2011, 3 years prior to the start of CMS-OP. Accordingly, we were unable to collect undisclosed payment data for this article’s study authors. Second, the CMS-OP database may have inaccuracies in its entries that are rarely corrected (27). One editorial reported that, as of 2015, the majority (72%) of disputed records within the database have gone unresolved (27). Finally, our sample may not be representative of all the research done on biologics and biosimilars in the management of IBD, as studies with neutral or non-significant results may have experienced publication bias and gone unregistered (28).

With these limitations in mind, we highlight the implications of our findings. First, academics and clinicians involved in both research and management of IBD should be cognizant of this high burden of undisclosed FCOI, as underreporting of conflicts may erode public trust in the research process (25). Although disclosure of FCOI has been increasing over the last decade (29), disclosure alone is likely insufficient in addressing bias related to FCOI. In particular, overreliance on disclosure may create the perception that a person is relieved of the responsibility of managing their conflict if disclosed (29, 30). Furthermore, the high burden of undisclosed FCOI we found may be attributed to our reliance on author self-reporting. In self-declaring their own conflicts, authors may not consider payments made several years ago or payments of a small dollar amount to warrant disclosure. To minimize bias, we recommend journals adhere to ICMJE guidelines to standardize reporting of conflicts, as well as to check author conflicts against publicly available databases for completeness of reporting.

## CONCLUSION

In summary, we found a high prevalence of total and undisclosed FCOI among authors of PSM studies comparing IBD biologics/biosimilars. We also found that studies with positive outcomes were significantly more likely to contain relevant author FCOI than neutral studies. PSM studies are an important means of biologic comparison in IBD. PSM studies in our cohort have been cited by CPGs and represent an important methodology that will be used increasingly in the future for IBD comparison. Accordingly, authors of PSM studies should ensure greater transparency with the reporting of industry relationships. Future research in this field should focus on developing a standardized methodology or regulations for the reporting of findings in PSM studies.

## Supplementary Material

gwac018_suppl_Supplementary_Material
