# Supplementary material for: Financial Conflicts of Interest in Propensity Score-Matched Studies Evaluating Biologics and Biosimilars for Inflammatory Bowel Disease
Source: J Can Assoc Gastroenterol. 2022 Jun 1;5(5):214–20. doi: 10.1093/jcag/gwac018 (PMC9527658; doi:10.1093/jcag/gwac018)
Supplement: gwac018_suppl_Supplementary_Material [file gwac018_suppl_supplementary_material.docx]

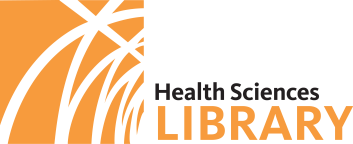


**Search Strategies & Documentation**

**Date search last run:** October 1, 2020

**Search limits: English**

Table of Contents

[Summary of the search results 1](#_Toc52523529)

[Search strategies 2](#_Toc52523530)

[Medline 2](#_Toc52523531)

[EMBASE 3](#_Toc52523532)

[Cochrane Library 5](#_Toc52523533)

# Table1: Summary of the search results

| Name of Database | Number of Hits | Notes |
| --- | --- | --- |
| Medline (Ovid) | 174 |  |
| EMBASE (Ovid) | 1218 |  |
| Cochrane Library | 274 |  |
| Total number of results with duplication | 1666 | Only Exact matched duplicated records were removed |
| Total Number of results after removing duplication | 1368 | Records are sorted by publication year and exported to an excel file |

#

# Table 2: Search strategies

## Medline

Database(s): Ovid MEDLINE: Epub Ahead of Print, In-Process & Other Non-Indexed Citations, Ovid MEDLINE® Daily and Ovid MEDLINE® 1946-Present

| # | Searches | Results | Annotations |
| --- | --- | --- | --- |
| 1 | exp Inflammatory Bowel Diseases/ | 81341 |  |
| 2 | (Inflammatory Bowel Disease* or Indeterminate colitis or undetermined colitis or Ulcerative Colitis or ulcerative proctocolitis or mucosal colitis or ulcerous colitis or colitis ulcerative or Colitis Gravis or colitis ulcerosa or Idiopathic Proctocolitis).ab,kf,ti. | 74734 |  |
| 3 | (Crohn Disease or Crohns Disease or Crohn's Enteritis or Regional Enteritis or Granulomatous Enteritis or Ileocolitis or Granulomatous Colitis or regional enterocolitis or Terminal Ileitis or Regional Ileitis or enteritis regionalis or morbus crohn or regional colitis).ab,kf,ti. | 48898 |  |
| 4 | Crohn?s Disease.ab,kf,ti. | 43424 |  |
| 5 | or/1-4 | 114111 |  |
| 6 | propensity score/ | 9585 |  |
| 7 | Comparative Effectiveness Research/ | 3593 |  |
| 8 | Comparative effectiveness.ab,kf,ti. | 8102 |  |
| 9 | "Average treatment effect*".ab,kf,ti. | 543 |  |
| 10 | (propensity adj3 (match or matched or matching or score or scores or scoring or analysis or weighted or weighting)).ab,kf,ti. | 28412 |  |
| 11 | or/6-10 | 38734 |  |
| 12 | Biological Factors/ | 6020 |  |
| 13 | Biological Products/ | 25348 |  |
| 14 | Immunosuppressive Agents/ | 96388 |  |
| 15 | exp Tumor Necrosis Factors/ | 153746 |  |
| 16 | exp Antibodies, Monoclonal, Humanized/ | 61281 |  |
| 17 | exp Infliximab/ | 10305 |  |
| 18 | anti?adhesion.ab,kf,ti. | 422 |  |
| 19 | (anti adj1 adhesion).ab,kf,ti. | 1067 |  |
| 20 | (Anti adj1 adhesive).ab,kf,ti. | 1195 |  |
| 21 | Anti?adhesive.ab,kf,ti. | 821 |  |
| 22 | (Anti adj1 integrin*).ab,kf,ti. | 864 |  |
| 23 | "Anti?integrin*".ab,kf,ti. | 20 |  |
| 24 | (Biologic* or tumor necrosis factor* or TNF* or Abciximab or Adalimumab or Alemtuzumab or Basiliximab or Bevacizumab or Brentuximab or Vedotin or Certolizumab or Cetuximab or Daclizumab or Denosumab or Gemtuzumab or Inotuzumab Ozogamicin or Ipilimumab or Natalizumab or Nivolumab or Omalizumabor Palivizumab or Panitumumab or Ranibizumab or Trastuzumab or Ustekinumab or vedolizumab or infliximab or golimumab).ab,kf,ti. | 1167718 |  |
| 25 | dt.fs. | 2238378 | the subheading for drug therapy |
| 26 | tu.xs. | 4773434 | subheading for therapeutic use |
| 27 | or/12-26 | 6105489 |  |
| 28 | 5 and 11 and 27 | 175 |  |
| 29 | limit 28 to english language | 174 |  |

## Table 3: EMBASE

Database(s): Embase Classic+Embase 1947 to 2020 September 30

| # | Searches | Results |
| --- | --- | --- |
| 1 | inflammatory bowel disease/ or exp crohn disease/ or exp ulcerative colitis/ | 161758 |
| 2 | (Inflammatory Bowel Disease* or Indeterminate colitis or undetermined colitis or Ulcerative Colitis or ulcerative proctocolitis or mucosal colitis or ulcerous colitis or colitis ulcerative or Colitis Gravis or colitis ulcerosa or Idiopathic Proctocolitis).ab,hw,ti. | 138886 |
| 3 | (Crohn Disease or Crohns Disease or Crohn's Enteritis or Regional Enteritis or Granulomatous Enteritis or Ileocolitis or Granulomatous Colitis or regional enterocolitis or Terminal Ileitis or Regional Ileitis or enteritis regionalis or morbus crohn or regional colitis).ab,hw,ti. | 105204 |
| 4 | "Crohn?s Disease*".ab,hw,ti. | 73512 |
| 5 | or/1-4 | 192447 |
| 6 | propensity score/ | 30891 |
| 7 | *comparative effectiveness/ | 9994 |
| 8 | Comparative effectiveness.ab,hw,ti. | 93612 |
| 9 | "Average treatment effect*".ab,hw,ti. | 680 |
| 10 | (propensity adj2 (match or matched or matching or score or scores or scoring or analysis or weighted or weighting)).ab,hw,ti. | 47151 |
| 11 | or/6-10 | 138531 |
| 12 | biological factor/ | 4927 |
| 13 | biological product/ | 45338 |
| 14 | immunosuppressive agent/ | 78989 |
| 15 | tumor necrosis factor/ | 140256 |
| 16 | exp agents used in inflammatory bowel disease/ | 311195 |
| 17 | exp monoclonal antibody/ | 585330 |
| 18 | exp monoclonal antibody/ | 585330 |
| 19 | exp tumor necrosis factor inhibitor/ | 91764 |
| 20 | anti?adhesion.ab,hw,ti. | 479 |
| 21 | (anti adj1 adhesion).ab,hw,ti. | 1315 |
| 22 | (Anti adj1 adhesive).ab,hw,ti. | 1472 |
| 23 | Anti?adhesive.ab,hw,ti. | 1003 |
| 24 | (Anti adj1 integrin*).ab,hw,ti. | 1194 |
| 25 | "Anti?integrin*".ab,hw,ti. | 50 |
| 26 | (Biologic* or tumor necrosis factor* or TNF* or Abciximab or Adalimumab or Alemtuzumab or Basiliximab or Bevacizumab or Brentuximab or Vedotin or Certolizumab or Cetuximab or Daclizumab or Denosumab or Gemtuzumab or Inotuzumab Ozogamicin or Ipilimumab or Natalizumab or Nivolumab or Omalizumabor Palivizumab or Panitumumab or Ranibizumab or Trastuzumab or Ustekinumab or vedolizumab or infliximab or golimumab).ab,hw,ti,tn,du. | 2287769 |
| 27 | dt.fs. | 3951193 |
| 28 | or/12-27 | 6171464 |
| 29 | 5 and 11 and 28 | 1229 |
| 30 | limit 29 to english language | 1224 |
| 31 | remove duplicates from 30 | 1218 |

## Cochrane Library

ID Search Hits

#1 MeSH descriptor: [Inflammatory Bowel Diseases] explode all trees 3312

#2 (Inflammatory Bowel Disease* or Indeterminate colitis or undetermined colitis or Ulcerative Colitis or ulcerative proctocolitis or mucosal colitis or ulcerous colitis or colitis ulcerative or Colitis Gravis or colitis ulcerosa or Idiopathic Proctocolitis):ti,ab,kw 7013

#3 (Crohn Disease or Crohns Disease or Crohn's Enteritis or Regional Enteritis or Granulomatous Enteritis or Ileocolitis or Granulomatous Colitis or regional enterocolitis or Terminal Ileitis or Regional Ileitis or enteritis regionalis or morbus crohn or regional colitis):ti,ab,kw 4776

#4 (Crohn?s Disease):ti,ab,kw 497

#5 #1 OR #2 OR #3 OR #4 9934

#6 MeSH descriptor: [Propensity Score] explode all trees 184

#7 MeSH descriptor: [Comparative Effectiveness Research] explode all trees 281

#8 Comparative effectiveness:ti,ab,kw 43929

#9 Average treatment effect*:ti,ab,kw 30036

#10 (propensity NEAR/3 (match or matched or matching or score or scores or scoring or analysis or weighted or weighting)):ti,ab,kw 2500

#11 #6 OR #7 OR #8 OR #9 OR #10 73250

#12 MeSH descriptor: [Biological Factors] this term only 44

#13 MeSH descriptor: [Biological Products] this term only 401

#14 MeSH descriptor: [Immunosuppressive Agents] this term only 5068

#15 MeSH descriptor: [Tumor Necrosis Factors] explode all trees 3424

#16 MeSH descriptor: [Antibodies, Monoclonal, Humanized] explode all trees 8761

#17 MeSH descriptor: [Infliximab] explode all trees 722

#18 (anti?adhesion or anti?adhesive or Anti?integrin*):ti,ab,kw 199

#19 (anti NEAR/1 (adhesion or adhesive or integrin*)):ti,ab,kw 169

#20 (Biologic* or tumor necrosis factor* or TNF* or Abciximab or Adalimumab or Alemtuzumab or Basiliximab or Bevacizumab or Brentuximab or Vedotin or Certolizumab or Cetuximab or Daclizumab or Denosumab or Gemtuzumab or Inotuzumab Ozogamicin or Ipilimumab or Natalizumab or Nivolumab or Omalizumabor Palivizumab or Panitumumab or Ranibizumab or Trastuzumab or Ustekinumab or vedolizumab or infliximab or golimumab):ti,ab,kw 71356

#21 #12 OR #13 OR #14 OR #15 OR #16 OR #17 OR #18 OR #19 OR #20 77397

#22 #5 AND #11 AND #21 274 (252 RCTS and 22 Sys rev)
